# Length of intervals between epidemics: evaluating the influence of maternal transfer of immunity

**DOI:** 10.1002/ece3.955

**Published:** 2014-01-28

**Authors:** Romain Garnier, Sylvain Gandon, Karin C Harding, Thierry Boulinier

**Affiliations:** 1 Centre d'Ecologie Fonctionnelle et Evolutive (CEFE), CNRS-UMR 5175Montpellier Cedex 5, F 34293, France; 2 Department of Ecology and Evolutionary Biology, Princeton UniversityPrinceton, New Jersey, 08544; 3 Department of Marine Ecology, Gothenburg UniversityBox 461, Gothenburg, SE-405 30, Sweden

**Keywords:** Epidemiology, harbor seal, host–parasite interactions, maternal antibodies, phocine distemper

## Abstract

The length of intervals between epidemic outbreaks of infectious diseases is critical in epidemiology. In several species of marine mammals and birds, it is pivotal to also consider the life history of the species of concern, as the contact rate between individuals can have a seasonal flux, for example, due to aggregations during the breeding season. Recently, particular interest has been given to the role of the dynamics of immunity in determining the intervals between epidemics in wild animal populations. One potentially powerful, but often neglected, process in this context is the maternal transfer of immunity. Here, we explore theoretically how the transfer of maternal antibodies can delay the recurrence of epidemics using Phocine Distemper in harbor seals as an example of a system in which epidemic outbreaks are followed by pathogen extinction. We show that the presence of temporarily protected newborns can significantly increase the predicted interval between epidemics, and this effect is strongly dependent on the degree of synchrony in the breeding season. Furthermore, we found that stochasticity in the onset of epidemics in combination with maternally acquired immunity increases the predicted intervals between epidemics even more. These effects arise because newborns with maternal antibodies temporarily boost population level immunity above the threshold of herd immunity, particularly when breeding is synchronous. Overall, our results show that maternal antibodies can have a profound influence on the dynamics of wildlife epidemics, notably in gregarious species such as many marine mammals and seabirds.

## Introduction

In vertebrates, the immune response to parasites relies on both a nonspecific innate response and a delayed and more specific acquired response (Frank 2002). Part of this acquired immune response relies on in the induction of immunoglobulins or antibodies, that can be transmitted to the offspring (Brambell 1970) through the colostrum and the milk in mammals or via the egg yolk in oviparous species. Because this transfer can result in a direct but temporary protection against the parasite (see for instance Wallach et al. 1992; Gustafsson et al. 1994), eco-epidemiological implications of this mechanism might be important (Gasparini et al. 2001; Grindstaff et al. 2003) but remain sparsely studied (Boulinier and Staszewski 2008).

Maternally transferred antibodies have, for instance, been shown to partly account for the seasonal dynamics of the infection in a vole-hantavirus system (Kallio et al. 2006, 2010). High levels of antibodies in mothers prior to reproduction result in a high proportion of temporarily protected newborns and, subsequently, a sudden reduction in the level of immunity in the population (when the concentration of maternal antibodies declines in the weaned offspring). Transfer of maternal antibodies is thus likely to modify population level immunity (Boulinier and Staszewski 2008), and the long term dynamics of epidemics (see for instance Guiserix et al. 2007). In systems where reproduction is synchronous and individuals aggregate during breeding season, the pulse of susceptible newborns can cause a rapid drop in the proportion of immune individuals (Roberts and Kao 1998), which can decline below the herd immunity threshold (Keeling and Rohani 2008) and favor the circulation of parasites on breeding grounds. Accounting for the passive protection of newborns would reduce the susceptible fraction of the population effectively susceptible to the parasite, a critical factor in the epidemiology of diseases (Stone et al. 2007), at a time when higher contact rates otherwise favor the onset of new epidemics (Loehle 1995). This mechanism may thus contribute to explain the long intervals between epidemics observed in some natural systems. It also represents an interesting interplay between life history characteristics, immunology, and eco-epidemiological dynamics.

A specific example in which such interactions might have been at play is the European harbor seal (*Phoca vitulina*) populations. They have endured two mass mortality events in 1988 and again in 2002, related to the circulation of a Morbillivirus, the Phocine distemper virus (PDV; Osterhaus and Vedder 1988; Jensen et al. 2002). Specific anti-PDV maternal antibodies have been reported long after the 1988 epidemic (Jensen et al. 2002) and up to 10 years after the 2002 epidemic in this population (Bodewes et al. 2013). The numbers of passively protected pups between 2002 and 2012 are consistent with the expectations of a classical epidemiological model. Using the same model, Bodewes et al. (2013) also show that the herd immunity in 2002 was not sufficient to prevent another epidemic from occurring, due in particular to the high turnover rate of the seal population. Earlier analyses of the PDV outbreaks do not include the effect of maternal protection on the herd immunity which may influence the intervals between epizootics. In both 1988 and 2002, the epidemics started in the same colony (Härkönen et al. 2006) which has led investigators to assume a rare introduction of the pathogenic agent on that colony (Grenfell et al. 1992; Harding et al. 2005b; Bodewes et al. 2013). The PDV might, however, have been introduced to the colony more often through, for instance, contacts with Grey seals (*Halichoerus grypus*) (Hall et al. 2006; Härkönen et al. 2006). In that case, herd immunity boosted by the passive protection of pups during the breeding season may have prevented the early re-occurrence of epidemics. The potential role of maternal antibody transfer in such stage-structured systems needs to be specifically explored (Klepac and Caswell 2011).

To investigate how the transfer of maternal antibodies could modify the recurrence of epidemics, we compared situations in which acquired immunity can or cannot be passively transferred to offspring. We built a model using realistic parameter values to describe the demography of a European harbor seal population and the epidemiology of the PDV and focused on the predicted intervals between epidemics. We investigated how the intervals between PDV epidemics can be influenced by maternal antibodies, the basic reproductive number of the pathogen, and by the synchrony of host reproduction. Finally, we investigated the effects of the transfer of maternal immunity in stochastic models where introduction of the pathogenic agent does not always lead to an epidemic.

## Materials and Methods

### Demography of the harbor seal/PDV model

We modeled an isolated harbor seal population using an age-structured Leslie model (Caswell 2001) parameterized as described by Härkönen et al. (2002; Table 1). The population is limited through density-dependent fecundities, typically the first life history parameter to be affected by limited food availability in marine mammals (Kjellqwist et al. 1995). The carrying capacity of the colony is fixed to 1000 females.

**Table 1:** Demographic parameters used in the Leslie matrix, following Härkönen et al. (2002). Fecundity are expressed in female pups by female. Survival is given as an annual survival probability.

Parameter	Value
Fecundity of females under age 4	0
Fecundity of females of age 4	0.17
Fecundity of females of age 5	0.33
Fecundity of females of age 6–26	0.47
Fecundity of females of age 27–37	0.35
Survival of pups of the year	0.75
Survival of subadults (age 1–4)	0.89
Survival of adults (age over 4)	0.95

Reproduction in harbor seals occurs once a year. To allow for the theoretical investigation of the birth synchrony on the PDV epidemiology, we allowed the degree of synchrony in the births to vary between 1 and 120 days. Synchrony was described by a parameter *σ*: when *σ *= 1, births are fully synchronous; when *σ* decreases, births are uniformly distributed over a maximum period of 120 days (no synchrony, *σ *= 0).

At the beginning of each simulation, the population structure is set to the stable age structure given by the Leslie model. The pathogen is then introduced (see below) and the effects of maternal antibodies in combination with birth synchrony, virulence, and stochasticity in the probability of epidemic spread are evaluated in terms of the resulting intervals between epidemics.

### Epidemiology of the harbor seal/PDV model

The epidemiological model assumes, for sake of simplicity, that individuals can be either permanently protected by their acquired immune response, temporarily protected by maternally transferred antibodies, or susceptible. The transfer of maternal antibodies to new cohorts of pups occurs even years after the mothers have been exposed to the virus for the first time (Jensen et al. 2002; Bodewes et al. 2013). The subsequent maternally acquired antibodies are supposed to last up to several months in harbor seal pups (Ross et al. 1994) and were set to last 120 days in the current study. This means that pups which receive maternal antibodies are protected during the reproductive season of the year, but susceptible the year after.

It has been demonstrated that PDV is not maintained in harbor seal colonies between epidemics (Swinton et al. 1998). We theoretically explore a case where the virus is introduced to harbor seals every year 10 days after the peak of the reproductive season. We first consider that the virus spreads efficiently each time (i.e., that the initial spread of the virus was sufficient to induce a full scale epidemic in an entirely susceptible population), and that immunity is the only driver of the epidemiological dynamics. Assuming a homogeneous mixing for simplicity, an epidemic can occur when the fraction of protected individuals falls below 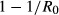, the threshold of herd immunity (Hethcote 2000) with *R*_0_ being the basic reproductive number (i.e., the number of seals infected by the first infected seal in a completely naive population). If herd immunity is sufficient, the virus cannot spread in the colony and all susceptible individuals remain susceptible the year after. On the contrary, if herd immunity is below the threshold, an epidemic occurs.

In a second analysis, we still introduce the virus each year, but assume the initial spread to depend on a probability of spreading. When herd immunity is above the threshold, this probability is set to 0. When immunity declines, the emergence probability is calculated following Lloyd-Smith et al. (2005) as 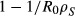 with *ρ*_*S*_, the ratio of susceptible individuals in the total population. Whether an epidemic occurred or not is then determined by a binomial challenge.

As shown by retrospective analyses of both the 1988 and 2002 epidemics, adults and newborns suffered increased epidemic mortalities compared to subadults (Heide-Jørgensen et al. 1992; Härkönen et al. 2007). We model this via an age-specific mortality as described in Harding et al. (2005b). All individuals surviving an epidemic were considered to have developed an acquired immune response and were therefore added to the pool of resistant individuals. This simplification is supported by observations revealing high exposure levels in the surviving females following the 1988 epidemic (Heide-Jørgensen and Härkönen 1992).

We explored the effect of different values of the basic reproductive number *R*_0_, considering published estimates, ranging from 2.03 to 2.8 (De Koeijer et al. 1998; Swinton et al. 1998; Klepac et al. 2009). We also explored how the interaction between the synchrony of births and the transfer of immunity influenced the predicted interval between epidemics.

## Results

**Figure 1:**
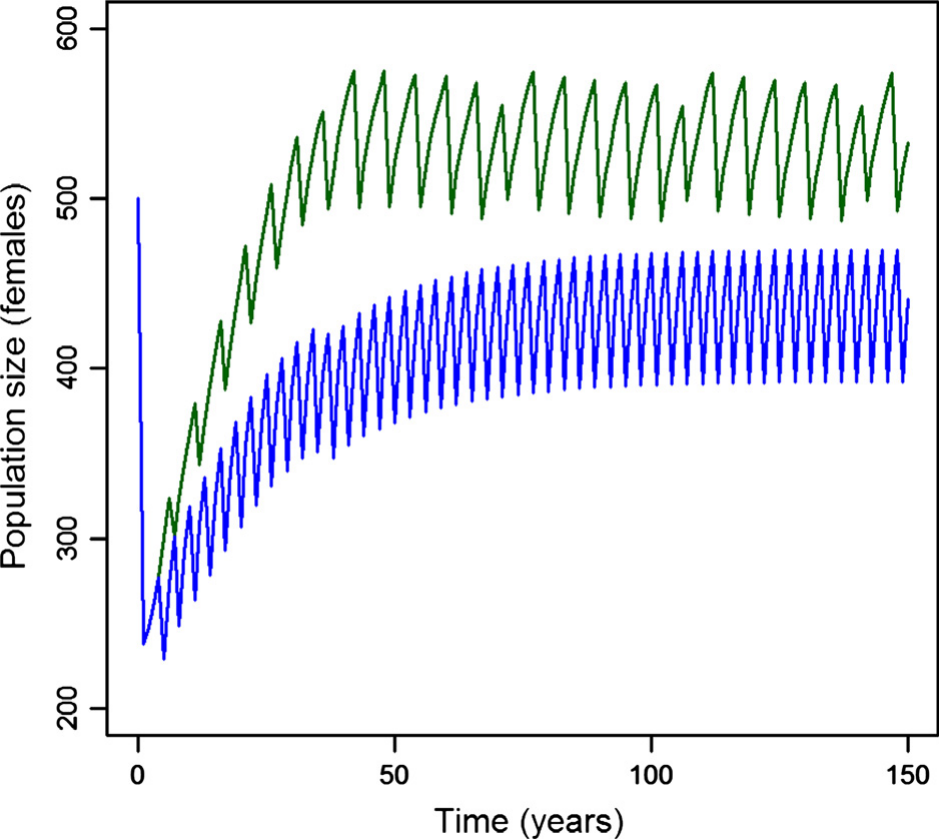
Population dynamics of an age structured model parameterized as for the Swedish harbor seal population enduring annual introduction of the Phocine Distemper Virus (PDV). Predicted dynamics show shorter intervals between PDV epidemics with acquired immunity only (blue line) compared to acquired immunity associated with maternal transfer of antibodies (green line). Reproduction happens synchronously, once a year on day 170 of the reproductive season and maternal antibodies are protective for 120 days. The pathogenic agent is introduced on day 180, and *R*_0_ = 2.8.

**Figure 2:**
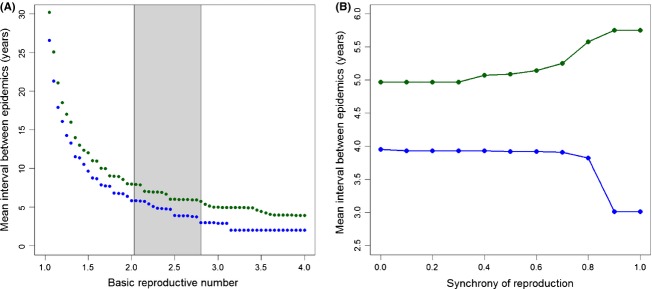
Effect of different parameters on the mean interval between PDV epidemics in a model parameterized for the Swedish harbour seal population and after 300 years of simulation when acquired immunity only protects adults (blue curves) or when it can be maternally transmitted to offspring (green curves). (A) Effect of the basic reproductive number (*R*_0_) on the predicted intervals between PDV epidemics. (B) Effect of the synchrony of the reproduction (*σ*) on the predicted intervals between PDV epidemics.

The transfer of maternal antibodies was found to be able to cause a significant increase in the predicted intervals between epidemics for PDV in European harbor seals (Fig. 1). Assuming a basic reproductive number of 2.8 (Swinton et al. 1998) and only acquired immunity produced intervals of 3 years between epidemics (blue curve). Allowing for the transgenerational transfer of immune protection increased intervals up to 6 years (green curve). The existence of a difference between the two scenarios did not depend on the exact value of the basic reproductive number (Fig. 2A). The mean interval between epizootics after 300 years of simulation was longer when the maternal transfer of antibodies was included for all assumed values of *R*_0_, although the difference was smaller for lower values of the basic reproductive number. Increasing *R*_0_ from 2.03 to 2.8, which are the boundaries of the published estimates based on the 1988 and 2002 epidemics (indicated by the grey segment in Fig. 2A), decreased the intervals between epidemics by about 2 years in both scenarios (with and without maternal protection of newborns).

How intervals between epidemics are influenced by the transfer of maternal antibodies also depend on the synchrony of reproduction (Fig. 2B). Increasing the synchrony of reproduction from low levels (0 to about 0.8) has almost no effect on the mean interval between epidemics. However, when synchrony is high and reproduction happens on a period of <24 days (*σ *> 0.8), a rapid response occurs when maternal antibodies can be transferred. In the model with maternal antibodies (green curve, Fig 2B), the interval between epizootics increases rapidly around that value of synchrony, while in the model without maternal protection, this interval suddenly decreases (blue curve, Fig. 2B). Variations in the timing of the introduction of the pathogen influence the effect of synchrony. When the parasite is introduced earlier in the season, higher synchrony may result in similar outcomes with or without maternal antibodies if the introduction occurs before the births begin.

**Figure 3:**
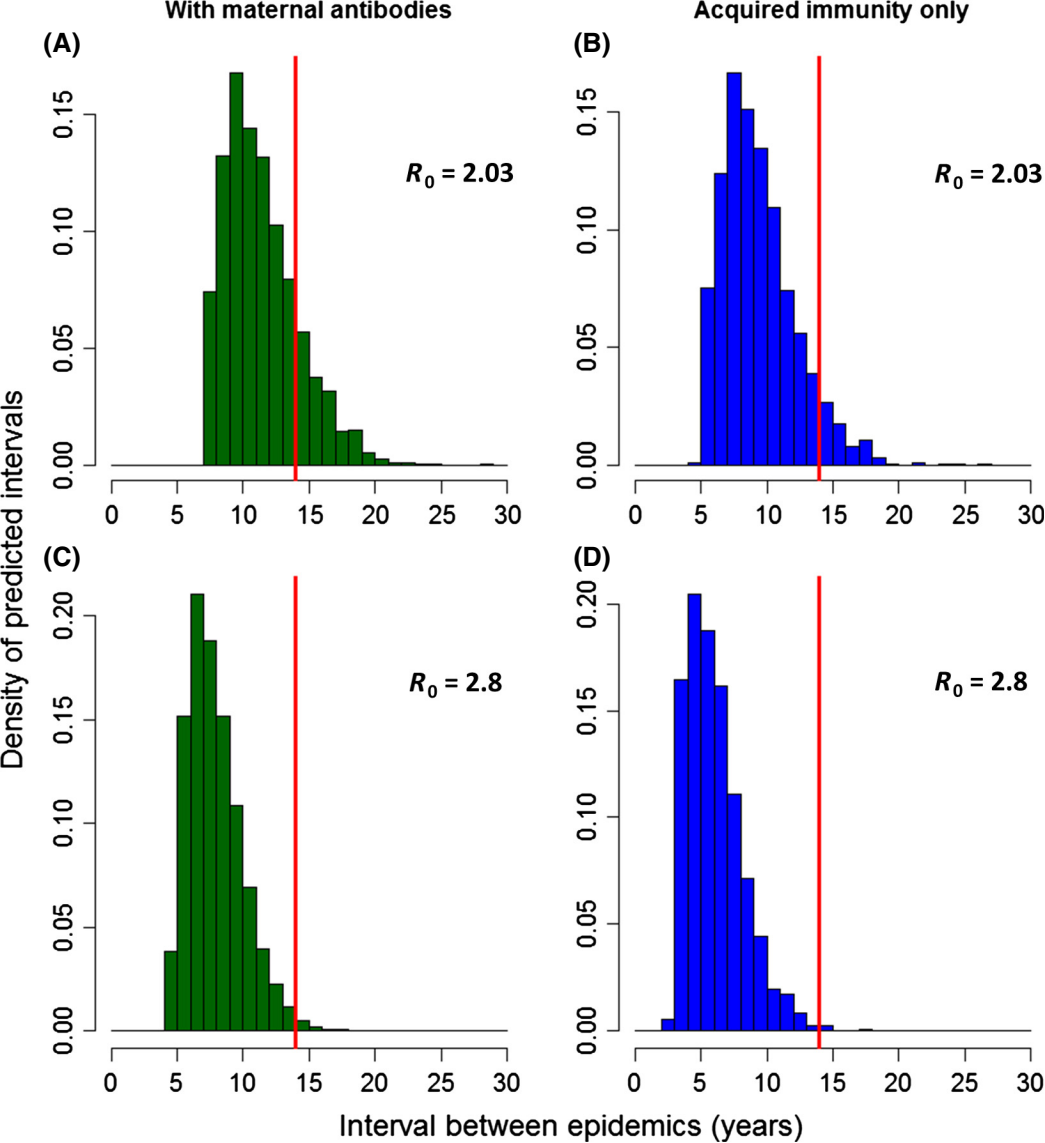
Distribution of the predicted intervals between PDV epidemics during a simulation of 20,000 years when the occurrence of an epidemic is stochastic. The model is parameterized for a Swedish harbor seal population in which acquired immunity can be transferred to newborns (i.e., with maternal antibodies in the green histograms) or is only protective for adults (i.e., without maternal anti bodies in the blue histograms). (A, B) Predicted distribution of intervals with *R*_0_ = 2.03. (C, D) Predicted distribution of intervals with *R*_0_ = 2.8. Births are synchronous (*σ *= 1) in all subplots. The horizontal red lines indicate the epizootic interval of 14 years as observed between 1988 and 2002 in the considered population.

Finally, when stochasticity is incorporated and PDV fails to spread every year in the population, the distribution of predicted intervals between epidemics is skewed towards even longer intervals when considering maternal immunity compared to only acquired immunity especially for low *R*_0_ (Fig. 3). This is most likely because the build-up of the immune fraction of the population by maternal transfer affects the probability of starting an epidemic even after the herd immunity threshold has been reached (thus the population escapes infection in some years where an epidemic would have happened in the deterministic model). As outlined earlier in the deterministic case, the difference between the two scenarios is robust over a range of *R*_0_ values. There is a difference of about 2 years between mean predicted intervals when *R*_0_ = 2.03 (Fig. 3A and B; mean_maternal antibodies_ = 11.87 ± 2.81 years; mean_acquired immunity_ = 9.86 ± 2.80 years) and when *R*_0_ = 2.8 (Fig. 3C and D; mean_maternal antibodies_ = 8.35 ± 2.08 years; mean_acquired immunity_ = 6.53 ± 2.13 years). In addition, the frequency of intervals of 14 years and above between epidemics is always higher when maternal antibodies are considered. This frequency is relatively low overall when *R*_0_ is closer to its highest estimate (with maternal antibodies: 2%, *n* = 48/2394 intervals; acquired immunity only: 0.5%, *n* = 17/3061 intervals). The frequency of long intervals increases substantially when *R*_0_ reaches its lower boundary (with maternal antibodies: 24.6%, *n* = 415/1684 intervals; acquired immunity only: 10.7%, *n* = 218/2027 intervals).

## Discussion

The transgenerational transfer of antibodies from a mother to her offspring temporarily reduces the proportion of susceptible individuals in the host population (Boulinier and Staszewski 2008). Our results indicate that this reduction can be relevant from an eco-epidemiological point of view as it can delay the initiation of a new epidemic. The extent of this delay depends on ecological factors specific to the host species such as age structure and the synchrony of reproductive events, but is not affected by the basic reproductive number *R*_0_ a key feature of the infectious agent. The modification of the dynamics of the susceptible fraction of the population, a critical epidemiological process (Stone et al. 2007), by the transfer of maternal antibodies could then partly explain the existence of long intervals between epidemics observed in some host-parasite systems.

The synchrony of the reproduction of the host has been shown to have important epidemiological consequences in wild systems, influencing for instance age-intensity curves (Cattadori et al. 2005), virus prevalence (Adler et al. 2008), or the local persistence of the pathogen in its host (Fouchet et al. 2008). We show here that synchronous reproduction may also amplify the epidemiological effects of the transfer of maternal antibodies. Interestingly, the value of synchrony above which maternal transfer of antibodies proves to be most important is well within the range of reproduction synchrony observed for harbor seals, in which 90% of births are concentrated on a 13–17 days period (Cordes and Thompson 2013). This effect of the synchrony of reproduction arises because synchronous reproduction enhances the peak of births before the infectious disease is introduced in the population. When newborns are mostly maternally protected (Fig. 2A, green curve), synchronous births increase the number of immune newborns present in the population at the time of exposure to the parasite thus strengthening the herd immunity and increasing the intervals between epidemics. Conversely, the reduction in the interval between epidemics when only acquired immune response is considered (Fig. 2A, blue curve) is the result of the dilution of population immunity by the birth pulse of susceptible newborns. This effect could be particularly important for colonial species. Indeed, in species such as seabirds or sea mammals where the reproductive season is constrained by environmental conditions such as water temperature (Harding et al. 2005a), food (Laidre et al. 2008) or breeding ground availability (Sundqvist et al. 2012), reproduction is limited to a brief temporal and spatial window of opportunity often resulting in high densities. The concentration of colonially reproducing individuals on spatially limited breeding areas can lead to high contact rates and exposure to parasites (Loehle 1995), but the transfer of protective immunity from mother to newborns may reduce the occurrence of epidemics while also providing the newborn with other immunological benefits (Grindstaff et al. 2003; Boulinier and Staszewski 2008; Hasselquist and Nilsson 2009; Garnier et al. 2012). Higher levels of transfer of maternal antibodies could thus be expected in colonially reproducing species.

In marine colonial species, the epidemiological effects of the maternal transfer of antibodies could also be amplified by specific temporal variations in the composition of the colonies, notably in relation to the age of individuals. It has, for instance, been shown that subadult harbour seals have a different behavior and do not interact as closely with other individuals as sexually mature seals and lactating pups (Härkönen and Harding 2001). This leads to a relative segregation of juvenile and adult harbor seals during the pupping season that could be related to a differential transmission pattern of PDV (Klepac et al. 2009). In seabirds, which are colonial and have delayed age at first reproduction, subadults can spend several years away from colonies before starting to prospect on colonies as part of the recruitment process (Reed et al. 1999). This low attendance of subadults on colonies observed in many colonial species would contribute to increase the effective immunity on the reproduction colonies and, in turn, to an even greater effect of the transfer of maternal immunity on ‘local’ herd immunity, hence resulting in longer intervals between epidemics.

Finally, our analysis focuses on how the spread of the infectious agent in the population is affected by the transfer of maternal antibodies, but a more detailed analysis would be required to understand how this mechanism could affect the size of the epidemics. Because a greater proportion of the population is susceptible when an epidemic occurs, delayed recurrence of a disease also results in epidemics of greater size (Harding et al. 2002, 2003; Bodewes et al. 2013). Recurrent epidemics of PDV have indeed been shown to have the potential to affect the local extinction risk of harbor seals if occurring at the observed frequency of 14 years (Harding et al. 2002), but accounting for the role of the transfer of maternal antibodies may mitigate this risk. Such models would ideally not be limited to an isolated colony, but rather consider a metapopulation of harbor seal colonies in Northern Europe (Harris et al. 2008). Interestingly, data on movement patterns of harbor seals are available (Dietz et al. 2013) and could be used to parameterize models of the spread of PDV depending on the immunity level of different subpopulations.

Infectious diseases can be a major threat for wildlife conservation (Daszak et al. 2000), in particular, in marine environments (Harvell et al. 1999). Our results suggest that the maternal transfer of antibodies, by temporarily protecting newborns in colonial breeding marine vertebrates, could have important consequences for the timing of epidemics and the dynamics of host–parasite interactions in such systems. It should, however, be kept in mind that little is known about the actual adaptive value of the maternal transfer of antibodies in natural populations (see Boulinier and Staszewski 2008 for a review). The protective role of maternal antibodies has indeed been shown clearly in some cases, but their role in natural systems is difficult to assess experimentally. More insights could be obtained by taking advantages of available datasets in domestic animals or even in humans (Leuridan and Van Damme 2007). Such datasets could, for instance, be used to parameterize models to understand the complex relation between maternal immunity and vaccinations (see, for instance, Metcalf et al. 2011). Carefully designed laboratory and field experiments also have the potential to increase our understanding of the role of maternal immunity in the wild (Garnier et al. 2013). For instance, it is only recently that a temporal persistence of maternal antibodies of several weeks after hatching has been reported for a particularly long-lived seabird species, the Cory's shearwater (Garnier et al. 2012). Our results also highlight that further studies integrating aspects of epidemiological and evolutionary ecology are needed to fully assess the potential role maternal antibodies may play in the dynamics of host–parasite systems involving colonial vertebrate species. This is notably important in the current context of global environmental changes and associated emergence of diseases in vertebrate populations (Keesing et al. 2010; Altizer et al. 2013).
